# Inflammation, immunity, and vascular remodeling in pulmonary hypertension; Evidence for complement involvement?

**DOI:** 10.21542/gcsp.2020.1

**Published:** 2020-04-30

**Authors:** Maria G. Frid, Joshua M. Thurman, Kirk C. Hansen, Bradley A. Maron, Kurt R. Stenmark

**Affiliations:** 1 University of Colorado, Denver, Anschutz Medical Campus, USA; 2 Brigham and Women’s Hospital, Harvard Medical School, USA

## Abstract

Pulmonary (arterial) hypertension (PH/PAH) is a life-threatening cardiopulmonary disorder. Experimental evidence suggests involvement of inflammatory and autoimmune processes in pathogenesis of PH/PAH, however the triggering and disease-promoting mechanisms remain unknown. The complement system is a key arm of innate immunity implicated in various pro-inflammatory and autoimmune diseases, yet, surprisingly little is known about the role of complement in PH/PAH pathogenesis. The preponderance of the existing data associates complement with PH/PAH via analysis of plasma and does not study the lung directly. Therefore, we aimed to resolve this by analyzing both the mechanisms of local lung-specific complement activation and the correlation of dysregulated plasma complement to clinical outcome in PAH patients. In our recent studies, reviewed herein, we show, for the first time, that  immunoglobulin-driven activation of the complement cascade, specifically its alternative pathway, in the pulmonary perivascular areas, is a key mechanism initiating pro-inflammatory processes in the early stage of experimental hypoxic PH (a form of “sterile inflammation”). In human patients with end-stage PAH, we have demonstrated that perivascular deposition of immunoglobulin G (IgG) and activation of the complement cascade are “longitudinally” persistent in the disease. We also showed, using unbiased network analysis, that plasma complement signaling, including again the Alternative pathway, is a prognostic factor of survival in patients with idiopathic PAH (IPAH). Based on these initial findings, we suggest that vascular-specific, immunoglobulin-driven dysregulated complement signaling triggers and maintains pulmonary vascular remodeling and PH. Future experiments in this area would facilitate discoveries on whether complement signaling can serve both as a biomarker and therapeutic target in PH/PAH.

## Inflammation in pulmonary vascular inflammation and remodeling

Pulmonary hypertension (PH) encompasses a group of severe clinical entities, including pulmonary arterial hypertension (PAH) where loss and obstructive remodeling of the pulmonary vascular bed is responsible for the rise in pulmonary artery pressure and pulmonary vascular resistance, resulting in progressive right heart functional decline and ultimately failure^1,2^. Patients typically present clinically when disease has become frankly symptomatic, and current treatments can ameliorate but not reverse disease progression. Therefore, a pressing need exists to understand the predisposing risk factors, initiating events, and the mechanisms of disease progression in order to improve early detection and therapy of this devastating syndrome.

At a National Institutes of Health Workshop in 2010, inflammation was acknowledged as an area of emerging interest, and the immune system was proposed as an essential contributor to the pathobiology of PH^3^. Since then, scientific focus on inflammation in PH has been increasing. In 2012 the Tuder group, in what is one of the few semi-quantitative studies in the modern era performed to assess pulmonary vascular remodeling, reported a significant correlation between perivascular inflammation and intimal and medial fractional thickness and a strong correlation with pulmonary artery pressure, the only two statistically significant correlations found in the study^4^. Further studies continued to link inflammation to the pathobiology of PAH^5,6^. Vascular inflammation can be associated with numerous pulmonary insults, including so-called “sterile inflammation”, which may arise in the context of environmental stresses, including hypoxia and sheer stress, in response to damage associated molecular patterns (DAMPs) released into the extracellular environment^7^. Moreover, chronic inflammation in PAH is associated with auto-immune forms of the disease^8^. However, the mechanisms triggering activation of the immune system and development of auto-immunity in PAH remain unknown.

There is strong evidence to suggest that inflammatory diseases of the vessel wall are largely orchestrated from the outside in^9–11^. Several groups, including ours, demonstrated that the vascular adventitia is a key site of immune activation^12–14^. It is increasingly appreciated that inflammatory responses are unique to the tissue where the inflammation occurs^9,15^. The idea has emerged that there is significant diversity in stromal cells, particularly in fibroblasts, and that function varies considerably among these subsets of cells, which have previously been lumped simply as “fibroblasts.” Our recent data suggest that one of the subsets of fibroblast-like cells that exists in the pulmonary hypertensive vascular wall is characterized by inflammatory cytokine production that exceeds that of other fibroblasts, SMCs, and ECs^14,16–18^.

Other fibroblast subsets exist that are functionally more similar to traditional myofibroblasts, while there are others that have anti-inflammatory properties. There is strong evidence that in the initial phases of PH in the animal models currently available, the earliest inflammatory responses occur in the adventitia^14^. In chronic persistent disease, this inflammation persists but then often involves both the medial and the intimal layers. This is consistent with the idea that in most normal arteries, the media is an immune-privileged site^19^.

Human studies clearly demonstrate that the most intense inflammatory responses in late-stage human PH are observed in the adventitia^12^. Thus, we posit that, although the nature of initial damage to the vascular wall can vary with different types of injuries in both the systemic and the pulmonary circulation, mounting evidence strongly supports the idea that peri-vascular inflammation may act as a driving force in the development of subsequent medial and intimal remodeling. Thus, it seems possible that inflammation represents a central mechanistic link between adventitial activation and vascular changes in response to a variety of stimuli. Elucidating the mechanisms contributing to this apparently microenvironmentally specific inflammation will be critical to understand inflammation and remodeling in PH.

## Complement: Overview

**Figure 1.**
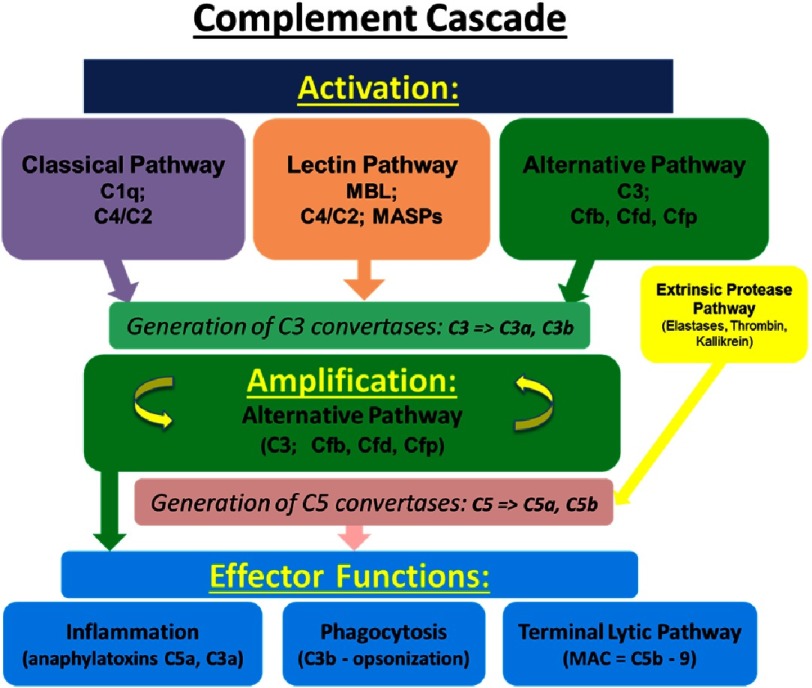
Schematic representation of complement cascade. Note that only a limited number of the components of each activation pathway is presented, and complement inhibiotors/regulators are not shown.

Complement is a cornerstone of innate immunity, playing a key role in host homeostasis, inflammation, and defense against pathogens and unwanted host elements. This system consists of a network of nearly 50 circulating, membrane-bound, and intracellular proteins and protein fragments activated by limited proteolysis in a cascade-like fashion (much like the coagulation cascade). Complement activation occurs via 3 major interconnected pathways (Figure 1):

(1) the classical, involving antibody-mediated activation via the C1 complex (comprising C1q as the main component), resulting in cleavage of C2 and C4 components;
(2) the lectin, triggered by carbohydrates (such as, for example, mannose-binding lectin) on cell surfaces, and resulting in cleavage of C2 and C4 components; and
(3) the alternative, which involves cleavage of C3 facilitated by complement factors B, D, and and stabilized by properdin^20–23^.

These pathways all converge onto formation of C3 convertases, which catalyze proteolysis of complement component C3 into the smaller C3a (anaphylatoxin) and the larger C3b fragment. C3b fragment associates with the C3 convertases to further form the C5 convertase that catalyzes the cleavage of complement component C5 into the smaller C5a (anaphylatoxin) and the larger C5b, which initiates the subsequent assembly of the membrane attack complex (C5b-9, MAC).

The numerous proteolytic complement fragments generated from the activation cascade provide crosstalk with other effector and regulatory systems, and functions as a bridge between innate and adaptive immune responses, interacting with several cell types, including macrophages, T- and B-cells, and platelets^20,21^.

Uncontrolled activation of the complement system can be very damaging to host tissues, and thus the complement cascade is very tightly controlled by soluble and cell membrane-bound regulators/inhibitors from the “regulators of complement activation” (RCA) family. These proteins act to avert the formation of convertase complexes on host cells and/or destabilize the preformed ones. Complement exerts functions well beyond those of detecting, killing, and removing invading microorganisms. For example, complement assists in the clearance of immune complexes, cellular debris, self-altered and apoptotic cells, and plays an essential role in pregnancy, CNS and other organ early development and in aging^24^.

This system is also involved in several non-immunologic processes, such as coagulation, angiogenesis, tissue repair, and generating a resistance to cell death, all of which have been linked to the pathogenesis of PAH^22,25^.

Collectively, these studies have demonstrated that complement is not a strictly intravascular system, but also one in which local secretion of complement components by tissue and infiltrating cells, and potentially even intracellular complement turnover contribute to complement-driven responses^25,26^. Recent work also supports the idea that locally or intracellularly generated complement fragments (anaphylatoxins, in particular) may have properties/functions that are different from their plasma counterparts. It is also important to note that cleavage of C5 complement can be achieved by an unconventional convertase-independent pathway (so-called “extrinsic protease pathway”) through other enzymes such as cathepsins, elastases, thrombin, and kallikrein^27,28^.

The diverse roles of complement have provided support for the concept that complement C3a and C5a anaphylatoxins participate in many of the classic processes that are hallmarks of cancer, as proposed by Hanahan and Weinberg^29^. This prompted several groups to examine the hypothesis that complement is activated in established tumors and pre-neoplastic lesions^30–32^.

Recent studies have revealed that complement activation can in fact promote the spread of tumors^33^. These effects are likely mediated through the anaphylatoxins C3a and C5a, produced during complement activation, acting on immune and inflammatory cells through specific cell surface receptors (C3aR and C5aR1/2). Further, it was found that C3a and/or C5a anaphylatoxins can exert profound influence on the tumor microenvironment by inducing a series of context-dependent changes, including: recruitment of tumor-promoting macrophages and CC-chemokine ligand 2 (CCL2) production^34^; a decrease in recruitment of CD4+ T-cells, neutrophils, and natural killer (NK) cells^35,36^; stimulation of a pro-tumoral phenotype for CD4+ T-cells and inhibition of IL10 expression by intramural CD8+ T-cells^37^; stimulation of pro-tumorigenic properties of mast cells and macrophages, including suppression of CD8+ T-cells cytotoxicity^28^; and promotion of pro-tumoral neutrophil extracellular trap (NET) formation^38^. However, work regarding the role of complement in tumor initiation and progression is far from over. Design of efficient complement-targeted therapeutics for cancer would require a better understanding of the mechanisms by which complement components contribute to tumor progression.

## Role of complement in pulmonary vascular inflammation and remodeling

Our and others’ work in humans with PAH and animal PH models recorded observations of cancer-like properties of cells from the hypertensive vessel wall, including excessive proliferation and apoptosis resistance, as well as early and persistent perivascular accumulation of monocytes/macrophages, augmented expression of pro-inflammatory cytokines/chemokines (GM-CSF, CCL2, CX3CL1, CXCL12, IL6), and immune dysregulation^11,12,39–44^.

However, until recently, little work has been performed to investigate the role of complement in the pathogenesis of PH/PAH. The preponderance of data, with the exception of a sole report^45^, associate complement with PAH via analysis of circulation^46–48^.

Until recently, this knowledge gap has cast uncertainty on the translational importance of complement to the pathogenesis of PAH. To begin to address this important issue, our group has recently worked to evaluate both the local lung-specific complement-driven processes and the correlation of dysregulated complement to clinical outcome in PAH patients.

### 1. Proteomic analysis of the complete matrisome in pulmonary arteries identifies complement and coagulation cascades as top upregulated pathways

There is intense interest in identifying key proteins involved in the PH disease process as they may provide potential targets of new therapies. The vast majority of work in this area has focused on changes in mRNA occurring during disease development using mRNA analysis of whole lung tissue. To our knowledge, there has not been a comprehensive study of the changes in the protein composition specifically of large or small pulmonary arteries in PH. We thus set out to perform a detailed analysis of the pulmonary vascular proteome in pulmonary hypertension.

Dr. Kirk Hansen has developed and optimized protocols for extraction of extracellular matrix (ECM) and matricellular proteins that have been refined using samples from a variety of sources, including tissue engineering studies^49–52^. He and colleagues have applied the protocols to samples based on cell culture, mouse tumors^53–56^ and patient tumors^55,57^ and over a dozen mouse organs^58–61^. Using these methods, Dr. Hasen’s laboratory has consistently identified the majority of ECM proteins (>99%) in both the soluble and, more importantly, insoluble fractions of the whole tissue extract.

**Figure 2.**
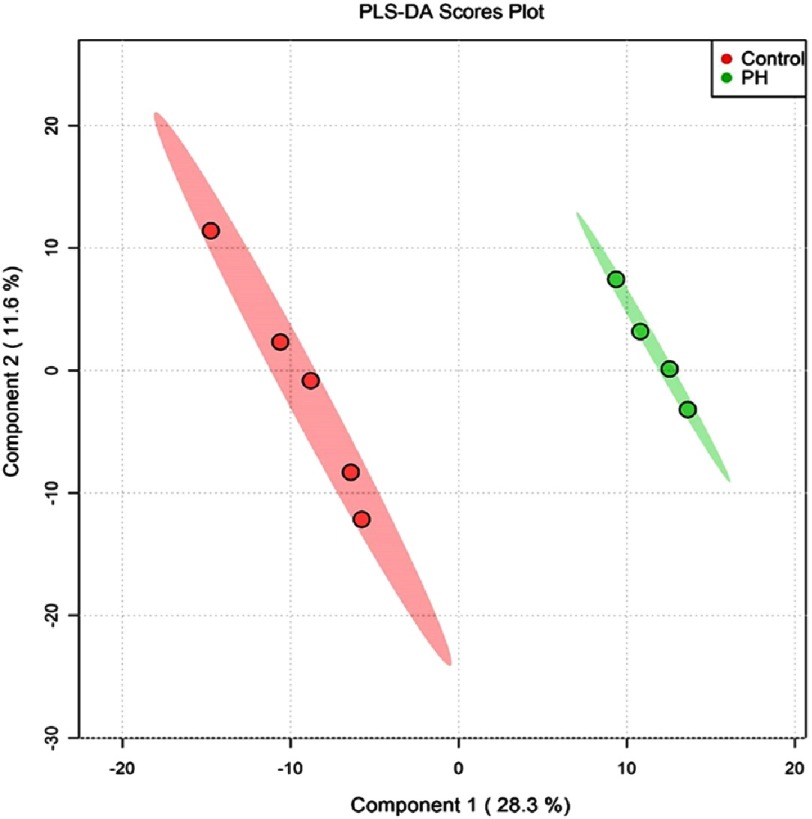
Proteomic analysis of whole distal pulmonary arteries from neonatal (15-day old) calves (control and chronically hypoxic) demonstrates profound differences in the proteome (PC analyses).

Quantification of the proteins, which has not previously been accomplished, was performed using a version of the quantitative concatamer (QconCAT) approach via a library of stable isotope-labeled (SIL, usually ^13^C _6_ - Lys or Arg) reporter peptides mixed with the samples before digestion^62,63^*.* Numerous ECM datasets collected in Dr. Hansen’s lab, as well as public data repositories have been utilized to select reporter “proteotypic” peptides for all observed ECM proteins, common cellular contaminants, proteins involved in ECM processing and internal standard proteins (850 total). Using these approaches, over 1300 proteins were examined in decellularized proximal and distal pulmonary arteries from control and hypoxic-hypertensive calves (an animal model of severe hypoxic PH)^64^ at 2 distinct time points in the PH disease process (early/14-days and late/16-months). Principal component analysis of the data demonstrated distinct differences between the control and PH proteasomes (Figure 2).

Interestingly, pathway analysis revealed that the most upregulated signaling pathway in the early stages of the disease process in both proximal and distal pulmonary arteries was related to activation of the complement and coagulation cascades (not shown). The data also demonstrate marked differences in the matrisome composition of different size vessels and at different times in the disease process. Whether these data are reflective of homogeneous change in the lung vessels or whether indeed there is heterogeneity within the proteasome related to specific type or degree of vascular remodeling remains to be investigated.

However, these observations stimulated us to pursue the role of complement in PH, specifically, when in the disease process and by what means complement was activated in the lung vasculature.

### 2. Vascular-specific complement activation in chronic experimental PH models and human PAH

**Figure 3.**
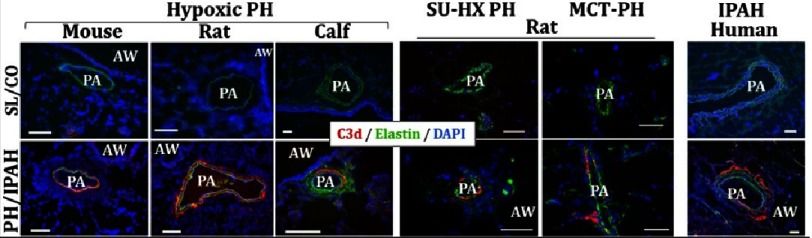
Activation of the complement cascade, as defined by deposition of C3d (terminal degradation fragment of C3 activation) is observed in a perivascular-specific pattern in the lungs of experimental animal models of PH (3-week hypoxic mice and rats; 2-week hypoxic calves; and rats with sugen-hypoxia (SU-HX)- or monocrotaline (MCT)-induced PH) and in humans with idiopathic PAH (IPAH). No deposition of C3d is observed in normal control lungs. Cryosectiones were immunolabeled with C3d-specific monoclonal antibody mAbs-C3d29 (**red** fluorescence), which distinguishes tissue-bound C3d from C3 or C3b. Pulmonary arteries (PAs) are visualized by autofluorescence of elastic lamellae (green). AW, airways. Cell nuclei are labeled with DAPI (blue fluorescence). Scale: 100 μm.

First, we searched to determine the evidence for local complement production and activation in experimental PH models and in human PAH. We evaluated localization of complement cascade activation via immunostaining for deposited C3d (the final activation/degradation fragment of complement C3, commonly accepted as a marker of activated complement cascade^65^), using a monoclonal antibody (mAb-C3d29) developed by Dr. Thurman and colleagues. This mAb specifically detects a covalently attached C3d fragment and not simply the presence of plasma-derived (“leaked”) C3 in the vessel wall^65^. C3d deposition was consistently observed in a perivascular-specific pattern in the lungs of mice, rats and calves with experimental chronic (2–3 week) hypoxic PH, and in rats with monocrotaline (MCT) PH and/or Sugen+hypoxia PH (Figure 3).

C3d deposition was also detected in pulmonary perivascular areas of IPAH patients, with evidence of occasional presence within the neointima of arteries with intimal thickening. C3d deposition in normal lung (rejected donors) was minimal to absent. Collectively, these data generated in different experimental PH models, at different stages of the disease process, and across species decisively demonstrated that complement activation is a longitudinal marker of PH/PAH. An important observation was of pulmonary perivascular-specific activation of the complement cascade.

Very little is known of the initiating mechanisms involved in the PH disease process due to lack of tissues at the very early stages of human disease. Therefore, we began a series of studies using experimental animal models, and specifically hypoxic rodents (mice and rats).

**Figure 4.**
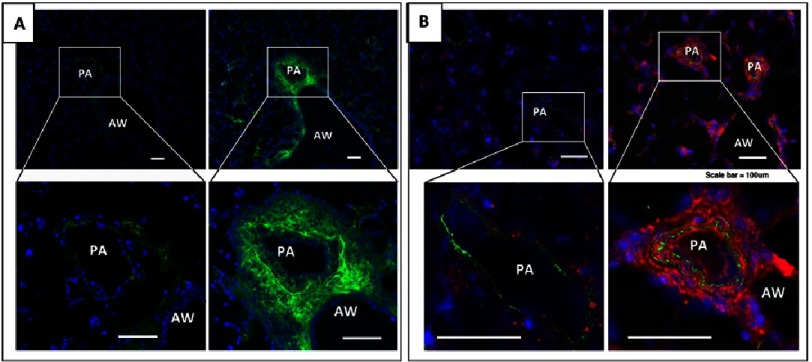
Hypoxia (3 day exposure) induces deposition of complement component C3 in pulmonary vascular-specific pattern in mice (A, green fluorochrome) and rats (B, red fluorochrome). PA, pulmonary artery; AW, airway. Scale, 100 μm.

### 3. Hypoxia causes activation of the complement cascade in a pulmonary vascular-specific manner

Short exposure of mice and rats to hypobaric hypoxia resulted in robust and vascular-specific deposition of complement component C3, suggesting *initiation* of activation of the complement cascade (Figure 4) (as of note, deposition of C3d fragment was not detected at this early time point). Increased numbers of perivascular cells expressing receptors for the anaphylatoxins C5a and C3a (C5aR1, C3aR1, respectively) was also observed and quantified.

**Figure 5.**
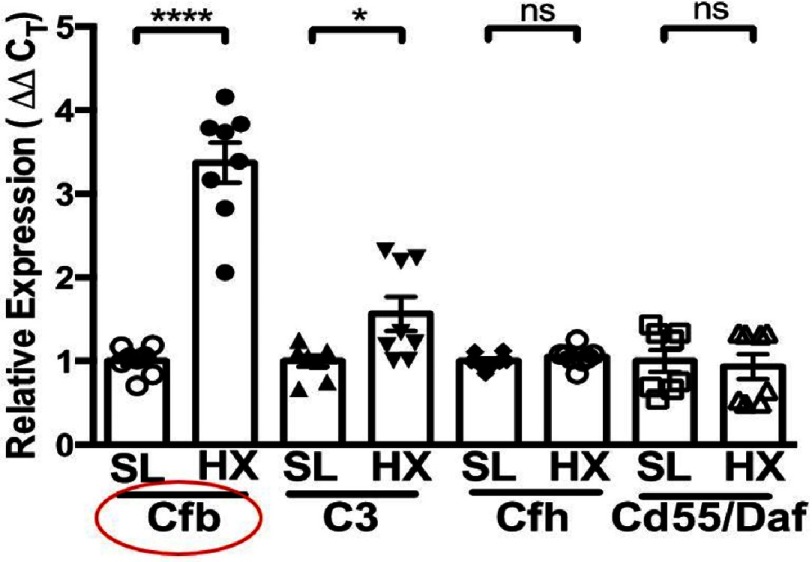
RT-PCR analysis of whole lung extracts from 3-day hypoxic (HX) wild-type (C57BL6) mice, compared to sea-levloe (SL) controls, demonstrates robust augmentation of complement factor B (Cfb), modest upregulation of complement C3, and no alterations in expression of complement inhibitors/regulators (complement factor H (Cfh), and Cd55/Daf), thus shifting the balance toward local activation of the alternative complement pathway. *P ≤ 0.05; ****P ≤ 0.0001; ns, not significant.

Furthermore, robust augmentation in the mRNA expression of complement factor B (Cfb, a key activator of the alternative pathway) was detected in whole lung extracts, with no significant changes in expression of complement inhibitors (i.e. complement factor H (Cfh) of the alternative pathway, and Cd55/Daf, decay-accelerating factor), thus shifting the balance toward activation of the complement cascade (Figure 5)^66^.

Collectively, these data provided evidence that the alternative complement pathway is a critical hypoxia-induced constituent in the initiation/early stage of hypoxic PH, in line with observations in other sterile inflammatory injuries^7,67^.

### 4. Hypoxia-induced pulmonary perivascular inflammation and cell proliferation are complement-dependent

First, we evaluated whether complement activation was involved in regulation of specific cellular/molecular events characteristic of the early stage of hypoxia-induced PH (monocyte/macrophage accumulation, cytokine/chemokine production and cell proliferation). To determine that, as well as specific complement components/pathways involved, we used mice genetically deficient in C3 (main component of the alternative pathway), Cfb (essential activator of the alternative pathway), and C5 (main component of the terminal/MAC pathway).

Perivascular accumulation of macrophages was assessed by immunostaining of lung sections for macrophage marker CD68 and via RT-PCR analysis of whole lung extracts. Mice deficient in Cfb (alternative pathway) and/or C5 (terminal /MAC pathway) were significantly protected from perivascular recruiment/accumulation of Cd68+ cells at 3-day hypoxic exposure. Intriquinly, and in contrast to the previously report^45^, C3-deficient mice exhibited a higly pro-inflammatory phenotype, which could be explained by other work demonstrating high level of anaphylatoxin C5a generation in C3-deficient mice due to thrombin production and C3-convertase independent cleavage of C5 complement^27^.

Because recruitment and activation of pro-inflammatory monocytes/macrophages in PH is known to be driven by chemokines/cytokines, such as Ccl2/MCP1 and Csf2/GM-CSF^68^, we next evaluated expression of these chemokines/cytokines in wild-type and complement-deficient mice. In 3-day hypoxic wild-type mice, expression of both Ccl2/MCP1 and Csf2/GM-CSF was robustly upregulated in pulmonary arteries and airways, as compared to sea-level wild-type controls.

Lungs of Cfb- and C5-deficient 3-day hypoxic mice demonstrated significant attenuation of Ccl2/MCP1 expression, and most remarkably, near complete abrogation of pulmonary Csf2/GM-CSF expression, which was confirmed both via RT-PCR analysis of whole lung extracts and RNAscope *in situ* hybridization in lung sections. The latter observation is of importance, as GM-CSF/CSF2 has emerged as a potent mediator of tissue, and specifically lung inflammation^68,69^. GM-CSF shows minimal levels in normal circulation, but can be locally produced at sites of inflammation, and promotes recruitment, pro-survival re-programming and pro-inflammatory activation of monocytes/macrophages, as well as participates in the induction of the inflammasome^70^.

Interestingly, among various myeloid types, only the inflammatory Ly6C^high^/Ccr2^+^ monocytes are vitally dependent on GM-CSF to instigate lung tissue inflammation and damage^70^, and a recent study reported increased numbers of these Ly6C^high^/CCR2^+^ monocytes in the blood and lungs of hypoxic mice^71^. Collectively these reports and our study demonstrate that hypoxia-induced pro-inflammatory monocyte expansion is dependent on GM-CSF signaling, which is in turn induced by local complement activation in the pulmonary vascular adventitia. Further support for these data comes from cell culture experiments using human pulmonary adventitial fibroblasts obtained from PAH patients. We evaluated GM-CSF responses to complement activation in these cells and observed that both mRNA and protein CSF2/GM-CSF expression was highly dependent on complement cascade activation via the alternative pathway.

We have additionally demonstrated that hypoxia-induced pulmonary perivascular cell proliferation, which is yet another feature of an early stage of PH disease process, is also complement (and, specifically, alternative and C5/terminal pathways)-dependent. Increased levels of perivascular cell proliferation, observed in 3-day hypoxic wild-type mice, were significantly attenuated in Cfb- and C5-dericient hypoxic mice.

Collectively, these observations will stimulate important future work to determine whether specific local tissue targeting of complement activation can interrupt the pro-inflammatory and pro-proliferative responses that are critical in PH initiation and progression.

### 5. Hypoxia-induced immunoglobulin deposition contributes to complement activation and perivascular pro-inflammatory, pro-proliferative processes

Having established an essential role of complement signaling in hypoxia-induced inflammation and cell proliferation, we set on identifying the upstream triggers of complement activation in this (hypoxic) model of sterile inflammation. As previous studies have suggested an important role of (auto)immune mechanisms in pathophysiology of PH/PAH^43^, we evaluated the contribution of immunoglobulins (Igs) to hypoxia-induced complement activation and vascular inflammation and remodeling.

**Figure 6.**
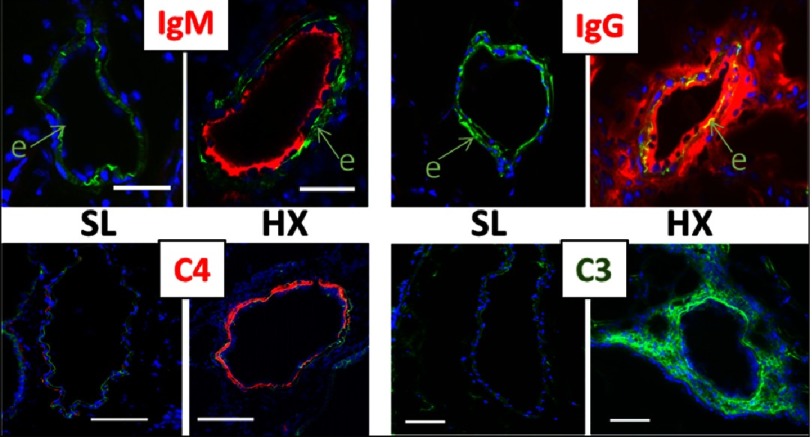
Early (3-day) hypoxia-induced deposition patterns of IgM and IgG are highly compartmentalized (luminal for IgM, and all vascular layers, with most prominent adventitial deposition, for IgG), and correlate with localization/deposition patterns of complement components C4 (classical activation pathway) and C3 (alternative pathway), respectively. SL, sea-level; HX, hypoxia.

Pulmonary vasculature of 3-day hypoxic rodents (mice and rats) demonstrated profound vascular-specific deposition of both IgM and IgG, yet in very distinct compartmentalized patterns. IgM deposition was restricted to luminal/medial vascular layers and correlated with deposition of complement C4 (the main component of the classical and lectin pathways), whereas deposition of IgG was detected in all vascular layers, with most prominent deposition in the perivascular adventitia, and correlated with deposition of complement C3 (the main component of the alternative pathway) (Figure 6).

**Figure 7.**
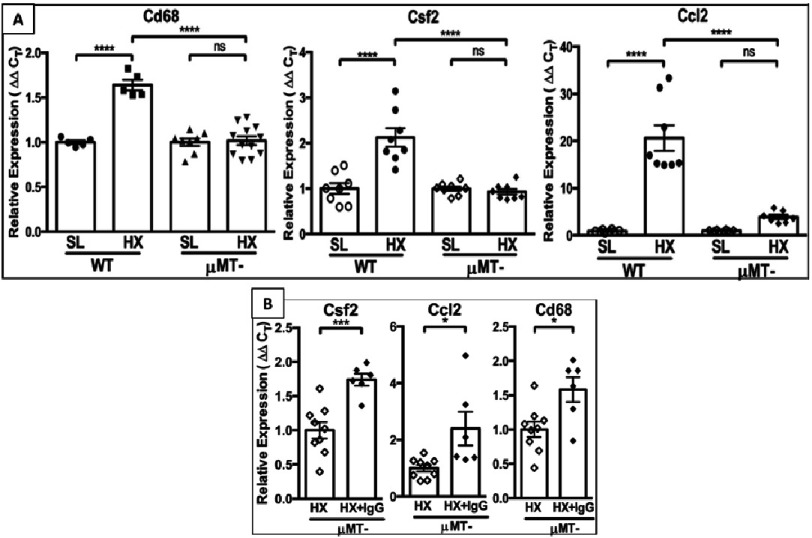
(A): RT-PCR analysis of whole lung lysates shows that immunoglobulin-deficient μMT- mice exhibit a “hypoxia-protected” phenotype (abrogated expression of macrophage marker CD68, pro-inflammatory cytokine Csf2/GM-CSF and chemokine Ccl2/MCP1) as compared to hypoxic wild-type (WT) mice. (B): Reconstitution of hypoxic μMT- mice with IgG (HX+IgG) restores a “hypoxic” pro-inflammatory phenotype as demonstrated via RT-PCR analysis of whole lung lysates.

Normal control lungs were completely devoid of IgM, IgG, C4, or C3. These observations led us to evaluate hypoxia-induced responses (complement activation and pro-inflammatory/pro-proliferative processes) in immunoglobulin-deficient μMT- mouse strain (deficient in all circulating Igs)^72^. Remarkably, μMT- mice demonstrated marked protection from hypoxia-induced vascular changes (no detectable complement activation; reduced accumulation of CD68+ macrophages and Ccl2/MCP1 expression; near complete abrogation of Csf2/GM-CSF expression; and significant decreases in cell proliferation) (Figure 7A).

Since all the examined hypoxia-induced processes (such as recruitment/accumulation of macrophages, C3aR/C5aR and cytokine/chemokine expression, and cell proliferation) occurred in perivascular adventitia of hypoxic mice and strongly correlated with deposition of IgG (but not IgM), we next performed experiments to determine whether reconstitution of hypoxic ⊠MT- mice with IgG to its normal circulating levels would reverse the observed protection. Using an IgG injection protocol, similar to that in human immunodeficient patients^73,74^, we observed restoration of the features of pathological hypoxia-induced “wild type-like” phenotype in ⊠MT- mice, including perivascular activation of complement (deposition of C3), increased accumulation of CD68^+^, C5aR1^+^ macrophages, upregulated expression of Ccl2/MCP1 and Csf2/GM-CSF, and increased cell proliferation (Figure 7B).

Collectively, these data indicate a central contribution of IgG to hypoxia-induced pro-inflammatory and pro-proliferative changes in the lung vasculature, and support the notion that, at least certain forms of PH, may be characterized by an immune-mediated component. Indeed, previous studies have reported detection of circulating autoantibodies in PH/PAH patients, as well as the presence of activated bronchus-associated lymphoid tissues in the lungs of patients with PAH^41,75^. However, specific mechanisms involved in this autoimmune dysregulation have not been explained and, specifically in hypoxic forms of sterile inflammation, remain unclear. Conventionally, the classical pathway of complement activation has been held responsible for inducing antibody-mediated complement activation, wherein pentameric IgM is more effective in activating complement than monomeric IgG.

However, it has been reported that certain IgG-immune complexes can activate the lectin pathway and have also been suggested to drive the alternative pathway^76,77^. It is important to note that the free C3b cleavage fragment (the main component of the C3 convertase) is short lived but becomes stable when covalently bound to certain IgG molecules or to cell surfaces, thus forming complexes that are more stable than C3b itself. In addition, tissue deposition of IgG may render the cell surface advantageous for alternative pathway propagation by diminishing the binding of the alternative pathway inhibitor, complement factor H (Cfh)^76^. Our data thus, when taken in context of other reports, support the idea that, at least in some immune complex-mediated PH/PAH cases, the alternative pathway can serve as a critical facilitator of inflammatory dysregulation –most likely in its role as a potent amplification loop, following initiation by the classical and/or lectin pathways.

Furthermore, the IgG/μMT- mouse reconstitution experiments led us to propose that, early in exposure to hypoxia, naturally occurring, so called “natural antibodies” (N-Abs), recognize and bind to neo-epitopes generated via hypoxic injury on resident pulmonary vascular wall cells. Natural antibodies are normally present in the circulation of healthy subjects in the absence of exogenous antigen stimulation (i.e. they are preexisting antibodies and can exert first response regulatory functions)^78^. At present, the identity of hypoxia-generated neo-epitopes recognized by circulating N-Abs is not known and is of interest.

**Figure 8.**
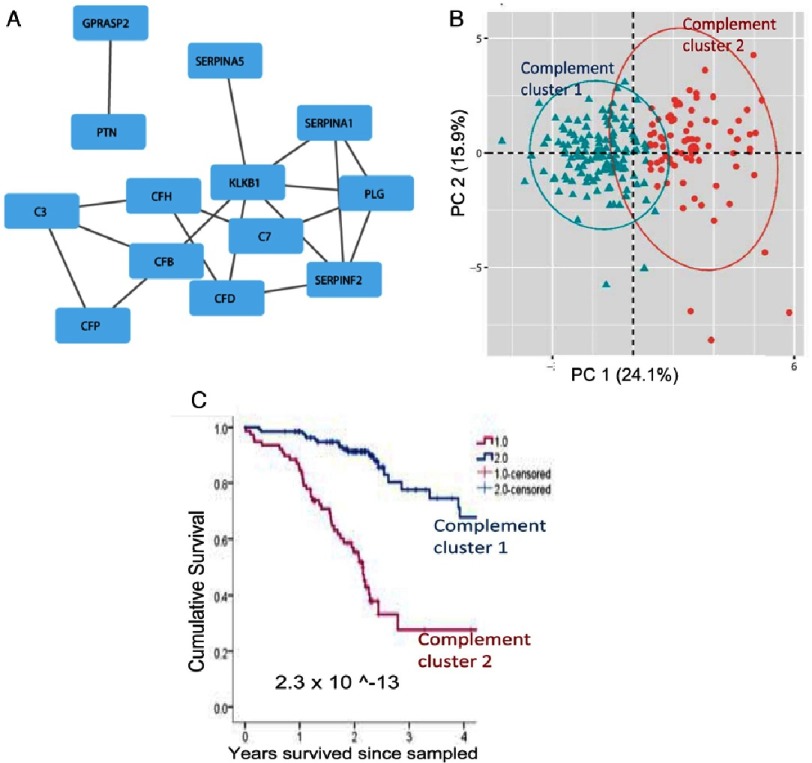
Plasma complement is a critical determinant of clinical outcomnes in patients with PAH. (A): Previously published data^46^, identifying circulating proteins with prognostic importance to PAH patients, were analyzed using a network medicine approach. Differentially expressed proteins were mapped to the consolidated human interactome resulting in a network that was enriched with complement pathway intermediaries, the *“complement-PAH network”* (13 proteins (blue boxes) and 18 protein-protein interactions (grey lines)). (B): Two distinct patient clusters were identified based on biological information derived solely from the *“complement-PAH network”*. Oval represents the estimated cluster boundaries determined by the patient data in each cluster. (C): Kaplan-Meier survival estimates in patients divided into two clusters (as shown in B) based on the plasma levels of proteins in the *“complement-PAH network”* are presented; thin vertical marks indicate where patients were censored during the time course.

### 6. Plasma complement is a critical determinant of clinical outcomes in patients with PAH

It has become increasingly clear that the PH pathobiology and even the contribution of inflammation to the PAH process is highly heterogeneous. Technologies are now available and allow advancement of studies focused on deep PAH phenotyping^79,80^. A recent study by Sweatt et al.^80^ demonstrated that blood cytokine profiles can distinguish PAH immune phenotypes with differing clinical risk that are independent of traditional WHO Group 1 subtypes. This study showed that these phenotypes could inform mechanistic studies of disease pathobiology and provide a framework to examine patient responses to emerging therapies.

**Figure 9.**
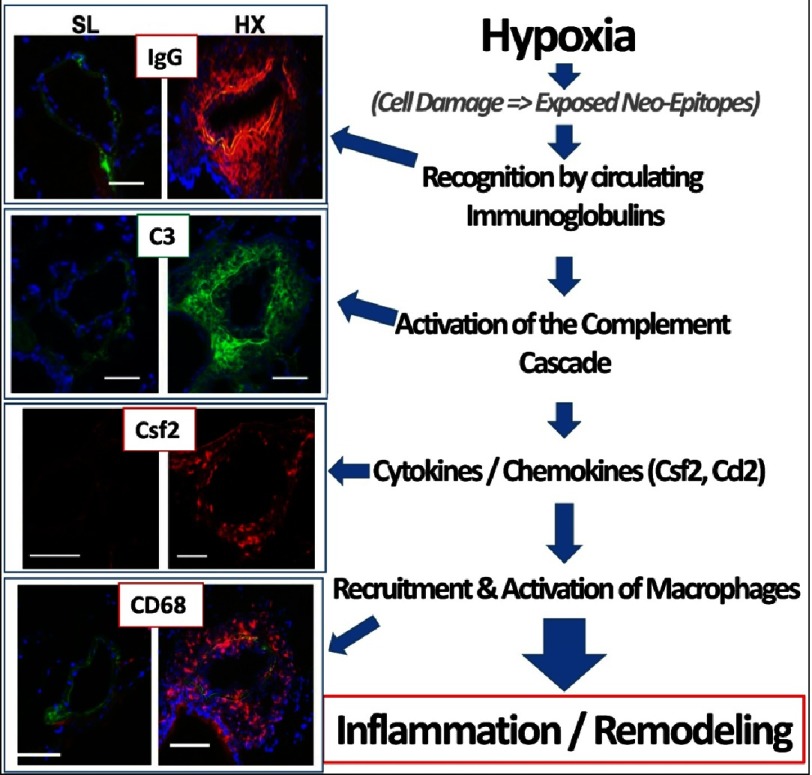
Schematic summary of the presented data, showing immunoglobulin (specifically IgG)-driven activation of the complement cascade, followed by production of pro-inflammatory cytokines/chemokines and recruitment of monocytes/macrophages, resulting in pro-inflammatory pulmonary vascular remodeling.

Another study by Rhodes et al. demonstrate that a panel of nine circulating biomarkers could predict, with some certainty, PAH disease progression^46^. Our observations, in collaboration with Drs. Brad Maron (Harvard University), Martin Wilkins (Imperial College of London) and colleages, support and expand these findings. Using network medicine to explore the integrated biological pathways that are important in PAH clinically, our findings demonstrate that clinical outcomes in PAH patients can be determined by a “complement-PAH network”. This name was assigned to the protein network network since it was heavily populated by complement intermediaries, particularly those of the alternative pathways (C3, Cfd, CfH, CfP, and C7 of the terminal (MAC) pathway) (Figure 8A).

The expression profile of proteins in the complement-PAH network alone was sufficient to identify two distinct patient subgroups (Figure 8B), which corresponded to significant and meaningful differences in the rate of all-cause mortality (Figure 8C).

These observations provide further data, which can be useful toward clarifying molecular mechanisms underpinning the role of complement in PH/PAH. The data in the human condition and its relevance to animal data were also validated by findings of almost identical perivascular-specific patterns of complement activation in the lungs of experimental PH animals and IPAH patients. Future studies will be needed to evaluate the influence of various types of complement activation on specific immune phenotypes in PH/PAH and, more importantly, to correlate blood and complement/immune phenotypes to the diversity of vascular lesions observed in PH/PAH, using the transcriptomic and proteomic approaches that are currently available.

## Conclusions and speculations

The presented and reviewed data establish, we believe for the first time, an essential causal role of immunoglobulin-driven complement activation in the early pro- inflammatory, pro-proliferative stage of hypoxic PH (form of sterile inflammation) (Figure 9). Dysregulated complement activation is also consistently observed in later stages of PH pathobiology in various experimental animal PH models, and appears as an important correlative determinant of clinical outcomes in PAH patients, thus comprising a persistent longitudinal determinant of PAH pathogenesis. Thus, therapeutic strategies utilizing targeted inhibition of local vascular-specific complement activation, and therefore persistent inflammation, may provide a novel approach for the treatment of certain patients with PAH. Because the current approaches for treatment of PAH in already symptomatic patients are limited to “managing” of the disease process, further discovery of novel prognostic targets in the asymptomatic pre-clinical period present an exciting new avenue in the development of more effective prevention and treatment strategies.
